# Citation analysis did not provide a reliable assessment of core outcome set uptake

**DOI:** 10.1016/j.jclinepi.2017.03.003

**Published:** 2017-06

**Authors:** Karen L. Barnes, Jamie J. Kirkham, Mike Clarke, Paula R. Williamson

**Affiliations:** a MRC North West Hub for Trials Methodology Research, Department of Biostatistics, University of Liverpool, Block F Waterhouse Building, 1-5 Brownlow Street, Liverpool L69 3GL, United Kingdom; b Centre for Public Health, Institute of Clinical Sciences, School of Medicine, Dentistry and Biomedical Sciences, Block B, Queen's University Belfast, Royal Victoria Hospital, Grosvenor Road, Belfast BT12 6BA, United Kingdom

**Keywords:** Core outcome set, Uptake, Citation analysis, Clinical trials, Systemic sclerosis, Rheumatoid arthritis, Eczema, Sepsis and critical care, Female sexual dysfunction

## Abstract

**Objectives:**

The aim of the study was to evaluate citation analysis as an approach to measuring core outcome set (COS) uptake, by assessing whether the number of citations for a COS report could be used as a surrogate measure of uptake of the COS by clinical trialists.

**Study Design and Setting:**

Citation data were obtained for COS reports published before 2010 in five disease areas (systemic sclerosis, rheumatoid arthritis, eczema, sepsis and critical care, and female sexual dysfunction). Those publications identified as a report of a clinical trial were examined to identify whether or not all outcomes in the COS were measured in the trial.

**Results:**

Clinical trials measuring the relevant COS made up a small proportion of the total number of citations for COS reports. Not all trials citing a COS report measured all the recommended outcomes. Some trials cited the COS reports for other reasons, including the definition of a condition or other trial design issues addressed by the COS report.

**Conclusion:**

Although citation data can be readily accessed, it should not be assumed that the citing of a COS report indicates that a trial has measured the recommended COS. Alternative methods for assessing COS uptake are needed.

**What is new?**

**Key findings**
• Core outcome set (COS) reports are mostly cited by publications that are not reports of trials.
• Not all trials citing a COS report have measured the outcomes in the COS.

**What this adds to what was known?**
• Citation analysis does not provide a reliable assessment of COS uptake by trialists.

**What is the implication and what should change now?**
• An efficient method to assess COS uptake based on current data needs to be developed.

## 1 Introduction

Inconsistency in the outcomes measured and reported across clinical trials in the same health condition creates difficulties for those drawing on the data produced by those studies to make informed decisions about health care. Variation in the outcomes selected hinders efforts to synthesize evidence from different trials and allows the potential for outcome reporting bias, the reporting of a subset of the outcomes measured based on results [1].

To help overcome these problems, the use of an agreed standardized set of outcomes, a core outcome set (COS), is recommended [2]. A COS consists of those outcomes considered to be most relevant for a specific health condition, typically agreed through consensus by a group of key stakeholders, that should be measured and reported, as a minimum, in all clinical trials of that condition [2]. Improving the consistency of the outcomes measured across trials by using a COS allows evidence from trials to be synthesized, encourages the reporting of all outcomes and, by incorporating outcomes that are most relevant to key stakeholders, ensures that the outcomes measured include the most appropriate.

A systematic review published in 2014 [3] identified 250 reports relating to 198 COS, and a recent update of this work found that the figure had increased to 227 COS by the end of 2014 [4]. As it is evident that a large number of published COS exist, with more than 100 also in development, it is important to assess their uptake by clinical trials. The continued development of COS that are not subsequently used in clinical trials will contribute to, rather than reduce, waste in research resulting from funding and time being invested in an initiative that is not then implemented. Furthermore, trials, and the eventual users of the reports of those trials, will not realize the benefits that using a COS can provide.

Assessing uptake also provides an opportunity to review and consider revision to a COS where it is evident that a particular outcome in the COS is not being measured or trials are consistently measuring an outcome that is not already included in the COS. Identifying COS with low uptake provides an opportunity to address barriers and facilitators to uptake.

Previous work has assessed the uptake of COS by examining trial reports included in systematic reviews, or identified through literature searches, to establish whether the outcomes in the COS were measured in the trials [5], [6], [7]. This method has proved to be resource intensive, and because the trial report will have been published several years after the trial's design stage when outcomes were chosen, it does not provide an up to date assessment of the uptake of a COS. More recently, a study used citations of the COS report to identify reports of trials that may have measured the COS [8]. As with other previous work, this study examined each individual trial report to assess uptake of the COS making this a lengthy method. However, citation analysis has the potential to provide an efficient method for COS uptake assessment if the number of citations received by a COS report alone could be taken as an indicator of the uptake of the COS.

The aim of our study is to examine the reliability of using citation analysis as a method of measuring COS uptake. In any scientific publication, an author will acknowledge the work of others by providing a reference to their publication. The publication referenced receives this acknowledgment in the form of a citation [9]. Citation analysis involves counting the number of citations received by a publication or author and taking this figure as a surrogate measure of impact [10], that is, the higher the citation count, the greater the impact. Citation analysis has the potential to provide an efficient method to assess the uptake of a COS if the number of citations received by a COS report could be reasonably taken as a surrogate measure of its uptake by trialists.

## 2 Methods

### 2.1 Selection of a citation analysis tool

For this study, we selected Scopus as the citation analysis tool. This is in keeping with a review of Scopus, Web of Science, and Google Scholar for citation analysis, which found that Scopus includes a wider range of journals, and in a search for a specific publication, Scopus retrieved 20% more citing articles than Web of Science. It was reported that the accuracy of Google Scholar was inconsistent [11].

### 2.2 Identification of COS reports

Of the 250 COS reports identified in the original systematic review [3], 173 reports, those published in 2009 and earlier, were considered for our citation analysis. This cutpoint was chosen to allow sufficient time for trialists to become aware of the COS, implement it in their study, and cite the COS report in the report of their trial by the time of the current analysis.

### 2.3 Selection of COS for citation analysis

Four COS were initially selected to evaluate the suitability of citation analysis for COS uptake assessment. Each COS provided a different aspect of interest to be investigated. A fifth COS was subsequently added to investigate a hypothesis suggested by the first round of evaluation. Appendix A at www.jclinepi.com details the characteristics of each COS.

#### 2.3.1 Systemic sclerosis

Systemic sclerosis was selected as a test case to assess the methods to be used for citation analysis and the identification of trials from the citations retrieved. There are just two COS reports for this condition [12], [13], neither of which are highly cited, and so the full process could be trialled from start to finish relatively quickly. One of the COS reports focused specifically on outcomes, whereas the other considered outcomes along with other trial design issues.

#### 2.3.2 Rheumatoid arthritis

Rheumatoid arthritis was selected as this health condition has one of the most recognized COS that was first published in 1993 following the 1992 OMERACT (Outcome Measures in Rheumatology http://www.omeract.org/) conference. The COS is reported in seven publications [14], [15], [16], [17], [18], [19], [20] all of which were included in the analysis, along with three other publications reporting earlier suggestions of COS for the condition [21], [22], [23]. All ten of the COS publications focused specifically on recommendations for outcomes. Another reason for the selection of rheumatoid arthritis was that the uptake of the OMERACT COS has been previously assessed by examining trial reports included in Cochrane reviews [5] to determine whether the outcomes in the COS had been measured. We wanted to establish how many of the trial reports that the previous study had found to have measured the COS would have been retrieved using citation analysis. If only a proportion of these trial reports were retrieved this would suggest that not all trials measuring a COS cite a COS publication in their trial report thus bringing into question the scope of citation analysis in identifying trials that had measured a COS.

#### 2.3.3 Eczema

Eczema was selected as this condition has a well-known COS due to a collaborative group working specifically on the agreement of COS in dermatological conditions, CSG-COUSIN (https://www.uniklinikum-dresden.de/de/das-klinikum/universitaetscentren/zegv/cousin/), which is linked to the Cochrane Skin Group. One report relating to the development of a COS for eczema was published before 2009, and its focus was on recommendations for outcomes. The report identified 20 outcome measurements and recommended that only three of these should be used for future studies. We assessed the uptake of the study's recommendations [24].

#### 2.3.4 Sepsis and critical care

Sepsis and critical care was selected as there are two associated COS reports, one of which specifically focused on the selection and measurement of outcomes [25], whereas the other considered outcomes while addressing other clinical trial design issues [26]. As with the systemic sclerosis example, identifying the difference in citations of these two COS reports may offer some insight into which type of publication is more likely to be accessed and to have its recommendations implemented by trialists.

#### 2.3.5 Female sexual dysfunction

Following the analysis of the first four COS, female sexual dysfunction was added to the evaluation to further investigate the level of citation of a COS report that included recommendations for outcomes alongside recommendations for other trial design issues [27].

### 2.4 Process

#### 2.4.1 Citation analysis

Publications that cited at least one of the COS reports were identified using Scopus, and the references to these publications were exported into Microsoft Excel. Duplicate entries of the same reference, caused by a publication citing more than one COS report, were removed to ensure that a reference to a COS report was not counted more than once. A publication relating to rheumatoid arthritis that had cited both the Felson et al. [14] and Boers et al. [17] COS reports, for example, would count as one citation for the COS.

#### 2.4.2 Identifying RCTs

Cochrane CENTRAL (Cochrane Central Register of Controlled Trials http://www.cochranelibrary.com/) was used as a tool to identify which of the citing publications were reports of trials. CENTRAL provides access to reports of randomized and quasirandomized controlled trials obtained from a variety of published and unpublished sources. Keywords, such as condition, intervention, and patient population, from the title of each citing publication were searched under the “record title” option in CENTRAL. Because CENTRAL contains publications other than trial reports, for example, systematic reviews, the abstracts of the articles identified by CENTRAL were screened to verify whether they were reports of trials. The full papers of those identified as trial reports following the abstract check were obtained for further investigation into why the COS report was cited and whether the outcomes in the COS were measured, to determine whether the citations received by a COS report could be reasonably attributed to trials measuring the COS. To confirm the eligibility of trial reports being included in the study, and the assessment of outcomes included in the trial by the reviewer (KB), the reports identified for rheumatoid arthritis were cross-checked with those identified in the previous rheumatoid arthritis uptake study. In cases where the same reports were identified the outcomes deemed to be measured by the trials were compared and there was 100% agreement between the findings of this and the previous study.

## 3 Results

### 3.1 Identifying trial reports from the citations

For each of the disease areas, the search of CENTRAL enabled publications listed in the citation report that were not reports of trials to be removed. The accuracy with which CENTRAL identified trials varied between disease areas (Table 1). CENTRAL was least accurate in identifying rheumatoid arthritis trials, with 126 (53%) of the 236 publications identified by CENTRAL confirmed as reports of trials. It was most accurate for eczema, with six (86%) of the seven records identified being confirmed as trial reports when the abstracts had been screened manually.

**Table 1:** Figures from citation analysis for each disease area

Disease name	Type of publication	No. of citations for COS reports	No. of citations identified as possible trials (CENTRAL)	No. of citations confirmed as trials (abstract check)	No. (%) of trials measuring all outcomes in COS	% of citations from trials
Systemic sclerosis	COS-only	27	1	0	0 (0)	0
	General design issues	97	15	10	0 (0)	10
Rheumatoid arthritis	COS-only	1,472	236	126	98 (78)	9
Eczema	COS-only	136	7	6	6 (100)	4
Sepsis and critical care	COS-only	64	4	4	1 (25)	6
	General design issues	711	23	13	0 (0)	2
Female sexual dysfunction	General design issues	723	40	23	6 (26)	3

*Abbreviation:* COS, core outcome set.

### 3.2 Number of trials identified that measured the COS

Not all of the citing publications that were identified as trials measured the recommended COS (Table 1). For three of the disease areas (systemic sclerosis, sepsis and critical care, and female sexual dysfunction), less than a third of the trials citing a COS report measured all the recommended outcomes. None of the 10 trials citing the report on general trial design issues for systemic sclerosis measured the COS, 25% of the four trials citing the COS-only report, and none of the 13 trials citing the general trial design issues report for sepsis and critical care measured the COS and 26% of the 23 trials citing the general trial design issues report for female sexual dysfunction measured the COS. In contrast, 78% of the 126 trials citing a COS-only report for rheumatoid arthritis measured all the outcomes in the COS and all six trials citing the outcomes-only report for eczema followed its recommendations on outcomes.

Assessment of a random sample of trial reports that had cited a COS report but did not measure all the recommended outcomes (a maximum of five reports for each condition and type of COS report, where available) identified a variety of reasons for citing the COS report (Table 2). Few of the trials were citing the COS report in relation to outcomes. The majority were referencing recommendations about other trial design issues that had been addressed in the COS report, for example, patient inclusion criteria, or definition of a disease or disorder. Some of the trials referencing a COS report that had focused only on outcomes had measured some of the COS outcomes and cited the report for this reason but did not provide an explanation for not measuring all of the recommended outcomes.

**Table 2:** Reasons that trials not measuring the COS cited COS reports

Disease area	Reason for citing COS report
Systemic sclerosis (general trial design issues report)^a^	Acknowledging that clinical trials are recognized to be difficult in the disease area
	Patient inclusion criteria (two trials)
	Patient population
	Determination of disease onset
Rheumatoid arthritis (COS-only reports)	Acknowledged COS but limited the number of outcomes to three that could be obtained by self-assessment by the patients
	The patient's global status and level of overall pain and the physician's global assessment were scored on a visual analogue scale.
	Measured the core set of measures apart from radiographs in trial lasting more than 1 year. The trialists acknowledged that this should be done in future trials.
	The variables chosen included 4 of 7 measures proposed for assessing disease activity by the ACR in 1993.
	Problems with outcomes have been addressed with the development of a COS
Sepsis and critical care (COS-only report)^b^	Named some of the proposed COS outcomes
	Inflammatory markers can provide additional support for a phase III study
	Sensitivity of organ dysfunction scales
Sepsis and critical care (general trial design issues report)	Definition of sepsis (four trials)
	Patient inclusion criteria
Female sexual dysfunction (general trial design issues report)	Definition of female sexual arousal disorder (three trials)
	Definition of hypoactive sexual desire disorder (two trials)

*Abbreviation:* COS, core outcome set.

a There were no citing trials for the systemic sclerosis COS-only report so no sample available.

b Three citing trials were available for the sepsis and critical care COS-only report that did not measure the COS.

### 3.3 Type of COS report cited in trial reports

For disease areas where two types of COS report exist, that is, reports focusing only on outcomes and reports considering outcomes while addressing other clinical trial design issues, the general design issue reports received more citations from trials (systemic sclerosis *n* = 10, sepsis and critical care *n* = 13) than the outcomes specific reports (systemic sclerosis *n* = 0, sepsis and critical care *n* = 4). However, none of the trials citing the general design issues papers measured the outcomes recommended. Although the COS-only reports for these disease areas also had a low number of citing trials measuring the COS (systemic sclerosis *n* = 0, sepsis and critical care *n* = 1), in cases where there was only one type of report, the outcome specific reports had considerably more citing trials that had followed their recommendations (rheumatoid arthritis 78%, eczema 100%) than a general design issues report (female sexual dysfunction 26%) (Table 1).

### 3.4 Comparison of methods of uptake assessment for rheumatoid arthritis trials

The previous study that examined trial reports included in Cochrane reviews to assess uptake of the OMERACT COS [5] found that 100 of the 350 trials that were identified measured all the outcomes in the COS. When the 350 trial reports were cross-referenced with those in the citation report, it was found that only 25 of these 350 trials had actually cited a COS report (Table 3). Therefore, for this particular sample of trials, citation analysis returned an uptake figure of 25 out of 350 trials, whereas 100 of these 350 trials had actually measured the outcomes in the COS. Further comparison of the trials shows that 20 of the 25 identified by citation analysis measured the outcomes in the COS.

**Table 3:** Comparison of uptake for 350 rheumatoid arthritis trials identified in Cochrane reviews

Trials identified and measuring the COS	Cochrane review method	Citation analysis method
No. of trials identified	350	25
No. (%) of trials that measured the COS	100 (29)	20 (6)

*Abbreviation:* COS, core outcome set.

### 3.5 Citations received from publications other than trial reports

For each of the disease areas we assessed, a large proportion of the citations received by the COS reports were from publications reporting on something other than trials (Table 1). For both types of COS report in all of the disease areas, at least 90% of the citations were not from reports of trials.

## 4 Conclusions

The aim of our study was to evaluate whether citation analysis would provide an efficient method to assess COS uptake. Although we have been able to demonstrate that citation data can be readily accessed, further investigation shows that it is not possible to assume that the citations received by COS reports are from trials measuring the COS they recommend. For example, of the 775 citations received by the sepsis and critical care COS reports, only 17 were from trials and of these 17 trials, just one measured the COS recommended by the report they had cited. These figures demonstrate first that COS reports are not only cited in trial reports but also in other types of publications and secondly that a trial report may cite a COS report for reasons other than adopting the COS, for example, other aspects addressed in the COS report such as patient inclusion criteria and definition of a disease. Therefore, it is not possible to use the number of citations received by a COS report alone as a surrogate measure for uptake of the COS by trials. Additional steps are required to generate an indication of uptake, as discussed below.

Further assessment of each citing publication was needed to establish whether it is a trial report and using CENTRAL to determine this can be a lengthy process. Keywords from each publication title needed to be manually input, and in cases such as the rheumatoid arthritis example, which has 1,472 records, this was time-consuming. As it is evident that CENTRAL contains publications other than trial reports, it is necessary to conduct further screening of abstracts to verify those publications identified in CENTRAL. The accuracy of CENTRAL to identify trials relies in part on the input of individual Cochrane Review Groups, and it is evident that there are differences in the maintenance of records and therefore the accuracy of CENTRAL between disease areas.

When all citing trials have been identified, an assessment of the trial report is needed to determine whether all the outcomes in the recommended COS were measured, adding a further time-consuming stage to the process as with other previous methods.

A recent study assessing uptake of The Prevention of Falls Network Europe (ProFaNE) COS for fall injury prevention [8] used citation analysis to identify trials citing the report of the COS. Similar to the disease areas reported here, a small proportion of the citations received by the COS report were from trials, with 34 trials found in 464 citations. The 34 trials were identified by screening the citing article's titles and abstracts and excluding reports that were protocols, pilot studies, or secondary reports of a trial already extracted. Most citing articles were observational studies (46%), editorials or reviews (23%), or methodological articles (13%). Analysis of the trial reports found that most trials made reference to the COS report in relation to other recommended methodology, for example, length of follow-up period, rather than outcomes. This finding is echoed in the study reported here, where COS reports are often referenced for design issues that had been addressed in addition to outcomes. Analysis of the ProFaNE COS also found that, although most trials had reported at least one of the recommended core domains, only one trial had reported on all core domains.

The results of the ProFaNE COS study support our finding that the number of citations received by a COS report cannot be taken as an indicator of its uptake and that further analysis is needed to ascertain whether the citing articles are trials measuring the COS. The number of steps and the amount of time needed to complete this process means that citation analysis is no more efficient for uptake assessment than the method of examining trial reports used previously [5], [6], [7]. Rather, citation analysis provides an alternative method of identifying trial reports that can then be assessed. Both citation analysis studies highlight that COS reports are mostly cited in articles that are not trial reports and it would be of interest to further investigate the types of articles citing COS reports and their reasons for doing so.

In addition to these findings, there are additional limitations of citation analysis for COS uptake assessment that should be noted.

Citation analysis does not take into account those trials that did measure the outcomes in a COS but did not cite a COS report thus affecting the accuracy of citation analysis as an indicator of uptake. As demonstrated by the comparison of methods to assess uptake of the rheumatoid arthritis COS, not all trials that measure a COS cite a COS report. This would lead to the rate of COS uptake being underestimated by citation analysis. In the case of the rheumatoid arthritis COS, a study previously demonstrated that 100 trials from a particular sample of 350 measured the COS [5]. However, when we cross-referenced this same sample with the citation report, only 20 of the trials that had measured the COS were present, plus a further five from the sample that had not measured the COS. Identifying a sample of trials and taking the number that had cited a COS report as a surrogate measure of COS uptake would remove the time-consuming process of examining the full trial report. However, this rheumatoid arthritis example demonstrates that uptake rate assessed in this way could be greatly underestimated. Regardless of which method is used to identify trials for assessment of COS uptake, whether through Cochrane reviews, literature searchers, or citation analysis, it is not possible to avoid a full examination of the trial report to obtain an accurate assessment of whether the outcomes in the COS had been measured. Therefore, it is necessary to consider ways in which examining trial reports can become more streamlined. This could be achieved if the format of trial reports allowed the outcomes measured in the trial to be clearly annotated so that it would not be necessary to read the full report to extract the information required.

A further limitation of citation analysis for COS uptake is the absence of data on the number of trials that were conducted in the relevant health condition for the time period being investigated. To make an accurate assessment of uptake it is necessary to know the proportion of the total number of trials that used the COS. Citation analysis can only retrieve information about trials that have cited a COS publication and does not provide the total number of trials conducted as a denominator.

Along with the evaluation of citation analysis as a method for assessing COS uptake, our findings raise an interesting hypothesis in relation to the effect that the focus of a COS report may have on COS uptake. Of the two COS reports for systemic sclerosis, one considered outcomes while addressing other clinical trial design issues [12], whereas the other focused specifically on the selection of outcomes [13]. Table 1 lists that none of the citing trials cited the outcomes specific paper and although this might be expected as that COS report was published in 2008 and the latest trial report identified was published in 2011, it generated a hypothesis that a COS recommended in a general clinical trial design publication is more likely to be implemented. However, further investigation shows that none of the trials citing the general clinical trial design paper measured the COS. They had cited the paper in relation to other design issues, for example, patient population and inclusion criteria, and not in relation to the choice of outcomes. Further investigation into the measurement of outcomes in a COS by trials that cited a general design issues publication for female sexual dysfunction revealed that 26% of trials citing the publication measured the COS. In contrast, 100% of trials that cited the eczema report and 78% of trials that cited a rheumatoid arthritis COS report, all of which focused specifically on the selection of outcomes, followed the report's recommendations on outcomes. It may be that when choice of outcomes is one of several issues addressed by a report, the recommendations relating to outcomes may become lost in the volume of information provided by the publication. Trialists looking specifically for advice on a particular area of trial design may overlook the recommendations regarding outcomes in these reports. Although reports focusing only on choice of outcomes may not be as highly cited as those covering a wider range of issues, the trialists citing these reports may be more likely to follow the recommendations in relation to which outcomes to measure. This may suggest that a COS should be reported independently of other design issues to attract the most attention, but this hypothesis requires further investigation.

It would also be of interest to further investigate which COS are measured in full, and compare their shared characteristics with those of COS that are only partially measured. This comparison may point to characteristics of COS that could affect their uptake in full. For example, none of the trials that cited the systemic sclerosis COS measured it in full. However, all of the trials measured at least one of the outcomes in the COS and the maximum measured in any one trial was six. Appendix A shows that this particular COS includes 14 outcomes and its partial use may be as a result of 14 outcomes being too many to include in a trial. Further investigation into fully and partially measured COS may indicate a maximum number of outcomes that should be included to enable trials to implement the full COS.

### 4.1 Future work

It is important to continue to consider efficient methods to assess the uptake of COS. Examining trial reports does not provide an up to date picture of COS uptake, and it is of interest to investigate methods that can identify outcomes that trialists are choosing for their studies at the present time, rather than those chosen at some time in the past. Clinical trial registries have the potential to provide an efficient assessment of COS uptake based on current information about the outcomes being measured in trials. A recent proposal suggested that trial registries could encourage trialists to record their use of a COS when registering their trial [28]. This information would provide a valuable resource allowing uptake of COS in ongoing trials to be assessed.
