# Supplementary material for: Citation analysis did not provide a reliable assessment of core outcome set uptake
Source: J Clin Epidemiol. 2017 Jun;86:153–9. doi: 10.1016/j.jclinepi.2017.03.003 (PMC5513440; doi:10.1016/j.jclinepi.2017.03.003)
Supplement: Appendix A [file mmc1.docx]

**Appendix 1 – COS characteristics**

|  |  |  | **Scope of COS** | | | **Development** | | |
| --- | --- | --- | --- | --- | --- | --- | --- | --- |
|  | **Link to other COS study** | **COS only or wider trial design issues** | **Population** | **Intervention** | **Intended use** | **Method(s)** | **Participants** | **Location** |
| **Systemic Sclerosis (SSc)** | | | | | | | | |
| **White 1995** | VD Steen author on both SSc reports | Wider trial design issues | Patients with diffuse cutaneous SSc of less than 24 months’ duration | Not specified | Phase III trials of disease-modifying interventions in SSc. | A committee analysed published and unpublished data and opinions from experts | Subcommittee of the Diagnostic and Therapeutic Criteria Committee of the Council on Research of the American College of Rheumatology | North America |
| Outcomes | 14 (death or survival time, weight or BMI, health status, physician global status, patient global status, serum creatinine, blood pressure, forced vital capacity, carbon monoxide diffusing capacity, left ventricle ejection fraction, serious arrhythmia requiring therapy, pseudo obstruction, malabsorption requiring total parenteral nutrition, skin score) | | | | | | | |
| **Khanna 2008** | Reference to White 1995 COS report | COS only | Not specified | Not specified | Observational and multi-centre clinical trials in SSc | 3-round Delphi exercise  and nominal group technique | Scleroderma Clinical Trials Consortium membership | North America, Europe, Asia, South America, Australia |
| Outcomes | 11 Domains, 31 parameters:  **Skin**: Modified Rodnan skin score (range 0–51), Visual analogue scale (VAS)/Likert score of patient’s global assessment for skin activity, VAS/Likert score of doctor’s global assessment for skin activity, Durometer. **Musculoskeletal**: Tender joint count, Tendon friction rubs assessed by the doctor, Serum creatinine phosphokinase aldolase. **Cardiac**: Cardiac echocardiogram with Doppler, Right heart catheterisation, 6-min walk test, Borg dyspnoea instrument. **Pulmonary**: Pulmonary function testing, Validated measure of dyspnoea, Breathing VAS from the Scleroderma Health Assessment Questionnaire (S-HAQ), High resolution computer tomography (HRCT) of the lungs: quantifiable scale. **Renal:** Calculated creatinine clearance based on serum creatinine (Cockroft–Gault or Modification of Diet in Renal Disease (MDRD) formula), Pre-defined renal crisis (presence or absence). **Gastrointestina**l: Body mass index (BMI), Validated gastrointestinal (GI) tract VAS scale (part of S-HAQ) or other SSc-validated GI questionnaire. **Health-related quality of life and function**: Health Assessment Questionnaire-Disability Index (HAQ-DI), VAS pain from the HAQ-DI, Short form-36 (SF-36) version 2. **Global health**: VAS/Likert patient global severity, VAS/Likert doctor global severity, Scleroderma-related health transition by patient, Scleroderma-related health transition by doctor. **Raynaud phenomenon**: Raynaud condition score, VAS Raynaud (part of S-HAQ). **Digital ulcers**: Active digital tip ulcer count on the volar surface, VAS digital ulcer (part of S-HAQ). **Biomarkers**: Acute phase reactant(s): erythrocyte sedimentation rate (ESR) and/or C-reactive protein (CRP). | | | | | | | |
| **Rheumatoid Arthritis** | | | | | | | | |
| **Bombardier 1982** | Co-author from OMERACT COS | COS only | Not specified | Not specified | Clinical trials | Nominal group technique | Rheumatologists, representatives of rheumatological societies, nurses, physiotherapists, researchers | North America |
| Outcomes | 11 (Joint count, pain relief, global assessment of change in disease activity, pain (patient related), global assessment of disease activity, morning stiffness, grip strength, self-care, physical activity/inability, pain (function), role activity) | | | | | | | |
| **Scott 1989** |  | COS only | Not specified | Slow acting anti rheumatic drugs | Clinical trials and individual patient management (practice) | Consensus meeting | 15 rheumatological workers from nine centres with special interests in the area of disease assessment | Europe |
| Outcomes | 5 (Mortality, morbidity assessment, functional index, drug reaction index, clinical and laboratory indices of disease activity) | | | | | | | |
| **van Riel 1992** |  | COS only | Not specified | Not specified | All international clinical trials | Results of study of the validity of 10 frequently used single variables and data from the literature | EULAR Standing Committee for International Clinical Studies | Europe |
| Outcomes | 7 (Number of tender joints, number of swollen joints, pain score, patient global score, C-reactive protein or erythrocyte sedimentation rate (ESR), health assessment, Larsen radiographic score) | | | | | | | |
| **Felson 1993** | Linked to OMERACT COS | COS only | Not specified | All interventions | All rheumatoid arthritis clinical trials | Literature review, nominal group technique, international conference | Experts in clinical trials and health services research, conference participants | North America, Europe |
| Outcomes | 8 (Tender joint count, swollen joint count, patient’s global assessment of disease activity, physician’s global assessment of disease activity, patient’s assessment of physical function, patient’s assessment of pain, laboratory evaluation of 1 acute-phase reactant, radiography or other imaging technique for trials lasting at least one year) | | | | | | | |
| **Fried 1993** | OMERACT COS | COS only | Not specified | Not specified | Rheumatoid arthritis clinical trials | Nominal group technique at international conference | Conference participants | North America, Europe |
| Outcomes | 8 (Tender joint count, swollen joint count, patient’s global assessment of disease activity, physician’s global assessment of disease activity, patient’s assessment of physical function, patient’s assessment of pain, laboratory evaluation of 1 acute-phase reactant, radiography or other imaging technique for trials lasting at least one year) | | | | | | | |
| **Tugwell 1993** | OMERACT COS | COS only | Not specified | Slow acting agents, nonsteroidal antiinflammatory drugs (NSAID), nonpharmacologic interventions | Rheumatoid arthritis clinical trials | Nominal group technique, plenary session with voting | Conference participants | North America, Europe, Australia |
| Outcomes | 8 (joint pain/tenderness, joint swelling, pain, patient global assessment, physician global assessment, disability, acute phase reactants, radiographs for studies of one year or longer) | | | | | | | |
| **Boers 1994** | OMERACT COS | COS only | Not specified | Antirheumatic drugs | Rheumatoid arthritis clinical trials | Direct questioning, rating of sample profiles of patients and trials, interactive voting and discussion | Conference participants (rheumatologists, methodologists, drug regulatory officials, pharmaceutical physicians) | North America, Europe, Australia |
| Outcomes | 8 (pain, patient global assessment, physical disability, swollen joints, tender joints, acute phase reactants, physician global assessment, radiographs of joints in studies of one or more years’ duration) | | | | | | | |
| **Kirwan 2003** | Linked to OMERACT COS | COS only | Not specified | Not specified | Rheumatoid arthritis clinical trials | Series of meetings and discussion sessions | Conference participants who had registered for the workshop including patients | Europe, North America |
| Outcomes | 8 (pain, patient global assessment, physical disability, swollen joints, tender joints, acute phase reactants, physician global assessment, radiographs of joints in studies of one or more years’ duration) plus recommendations to explore inclusion of subjective experiences of rheumatoid arthritis identified by patients, e.g. sense of wellbeing, fatigue, disturbed sleep | | | | | | | |
| **Kirwan 2005** | Linked to OMERACT COS | COS only | Not specified | Not specified | Rheumatoid arthritis clinical trials | Overview presentations, discussion groups and plenary sessions | Participants at the Patient Perspective Workshop at OMERACT 7 including 19 patients | Europe, Australia |
| Outcomes | 8 (pain, patient global assessment, physical disability, swollen joints, tender joints, acute phase reactants, physician global assessment, radiographs of joints in studies of one or more years’ duration) plus recommendation that fatigue be included | | | | | | | |
| **Kirwan 2007** | Linked to OMERACT COS | COS only | Not specified | Not specified | Rheumatoid arthritis clinical trials | Discussion groups in workshop | Participants at the Patient Perspective Workshop at OMERACT 8 including 20 patients from 10 countries and 60 other OMERACT participants | Europe, North America |
| Outcomes | 8 (pain, patient global assessment, physical disability, swollen joints, tender joints, acute phase reactants, physician global assessment, radiographs of joints in studies of one or more years’ duration) plus recommendation that fatigue be included | | | | | | | |
| **Eczema** | | | | | | | | |
| **Schmitt 2007** | Linked to HOME (Harmonising Outcome Measures for Eczema) COS | Outcomes only | Not specified | Therapeutic interventions for atopic eczema | Randomized controlled trials and clinical practice | Systematic review and survey of clinical experts and patients | Dermatology experts, patients, carers | Europe |
| Outcome recommendations | Only SCORAD, EASI and POEM perform adequately and should be used in future studies | | | | | | | |
| **Sepsis and critical care** | | | | | | | | |
| **Marshall 2005** |  | COS only | Not specified | Not specified | Sepsis clinical trials | Expert colloquium | Sepsis researchers, clinical epidemiologists, experts in the development and implementation of outcome measures in rheumatology, neurology and oncology | North America, Europe, Australia, Asia |
| Outcomes | 2 (mortality beyond 28 days, health-related quality of life) | | | | | | | |
| **Goldstein 2005** |  | Wider trial design issues | Age 0-18 years | Not specified | Clinical trials in pediatric sepsis | Consensus conference | 20 experts in sepsis and clinical research | North America, Europe |
| Outcomes | 2 (mortality, overall level of functioning) | | | | | | | |
| **Female sexual dysfunction** | | | | | | | | |
| **Basson 2000** |  | Wider trial design issues | Female | Not specified | Not specified | Delphi, Consensus conference | 19 experts in female sexual dysfunction | North America, Europe |
| Outcomes | 3 (specific changes in sexual function, personal distress, quality of life | | | | | | | |
